# Dual-Functional Cross-Meandering Resonator for Power Frequency Electromagnetic Shielding and Wireless Sensing Communication

**DOI:** 10.3390/s24175615

**Published:** 2024-08-29

**Authors:** Fengyuan Gan, Xiangshuo Shang, Xuelei Yang, Shuo Li, Yi Zhou, Wei Li

**Affiliations:** 1 School of Physical Science and Technology, Southwest University, Chongqing 400715, China; 2 National Key Laboratory of Materials for Integrated Circuits, Shanghai Institute of Microsystem and Information Technology, Chinese Academy of Sciences, Shanghai 200050, China; sxs3830@163.com (X.S.); yxl1231@mail.ustc.edu.cn (X.Y.); yizhou_scarlett@126.com (Y.Z.); 3 College of Energy and Mechanical Engineering, Shanghai University of Electric Power, Shanghai 201306, China; shuoli0107@gmail.com

**Keywords:** cross-meandering resonator, subwavelength, electromagnetic shielding, wireless sensing communication

## Abstract

The research on MEMS wireless sensing technology adapted to strong power frequency electromagnetic field environments is of great significance to our energy security, economic society, and even national security. Here, we propose a subwavelength cross-meandering resonator (0.49*λ*_0_ × 0.49*λ*_0_) to simultaneously achieve power frequency electromagnetic field shielding and wireless communication signal transmission. The element size of the resonator is only *λ*_0_/11, which is much smaller than that of previous works. In the resonator, a resonance mode with the significant near-field enhancement effect (about 180 times that at *f* = 1 GHz) is supported. Based on the self-made shielding box experimental setup, the measured shielding effectiveness of the resonator sample can reach more than 33 dB. Moreover, by integrating the cross-meandering resonator with the MEMS sensor, a wireless communication signal can be successfully transmitted. A dual-function cross-meandering resonator integrated with sensors may find potential applications in many military and civilian industries associated with strong power frequency electromagnetic fields.

## 1. Introduction

As the backbone of the energy Internet, the ultra-high voltage (UHV) power grid is developing toward intelligence, informatization, and digitization. With the rapid advancement of micro-electro-mechanical system (MEMS) wireless sensing technology for smart power grids, the demand for miniaturized electronic devices with various functions is increasing [1,2]. It is worth noting that UHV power grids are accompanied by strong power frequency (~50 Hz) electromagnetic fields, which bring out not only severe electromagnetic interference but also physical damage to electronic devices [3]. In addition to UHV power grids, many military and civilian industries, such as generator sets, large ship electromagnetic power systems, and aircraft carrier electromagnetic catapult devices, also suffer from wireless sensing technology problems in strong electromagnetic fields. Therefore, it is an urgent need to address strong electromagnetic field interference while maintaining reliable wireless communication with other devices or systems.

For power frequency electromagnetic shielding, Faraday cages [4], erecting shielding wire, shielding nets, and silver-clad copper-based electromagnetic shielding composite coatings [5] have been investigated. However, a large Faraday cage is difficult to use as the power frequency electromagnetic shielding of small electronic components [4]. As far as erecting shielding wire and shielding nets are concerned, the effect of power frequency electromagnetic shielding is not significant. The shielding effectiveness of composite shielding materials is also not as good as that of metal alloy materials [5]. Moreover, these methods cannot guarantee the wireless communication signal transmission of electronic sensing components. In recent years, the frequency selective surface (FSS) or microwave antenna structure has been utilized to achieve wireless communication [6,7,8,9,10,11,12,13,14,15,16,17,18,19,20]. For example, by combining parallel LC resonators [11] or cascaded hybrid resonant elements [13], a frequency selective surface (FSS) was designed to achieve wideband wireless communication [10,12]. However, the difficulty of miniaturization and fabrication limits the application of FSSs in wireless communication. By using one circular bottom patch and two circular top patches in an air suspended inverted microstrip configuration, a new microstrip antenna was designed to operate at a range of frequencies of 1.8–2.6 GHz [13]. Though the overall dimension of the antenna is much smaller than that of the FSS, the ratio of its dimension to resonant wavelength is 0.94, which is not sufficient for low-frequency band applications. More importantly, these works did not experimentally explore the shielding effectiveness of the structure on power frequency electromagnetic fields. Therefore, it is still a great challenge to simultaneously achieve power frequency electromagnetic shielding and low-frequency wireless communication using a miniaturized resonant structure.

In this work, a subwavelength cross-meandering resonator (0.49*λ*_0_ × 0.49*λ*_0_) was designed to experimentally demonstrate power frequency (50 Hz) electromagnetic shielding and wireless communication signal transmission. The resonant element (~λ_0_/11, λ_0_ is the free-space wavelength) is created by sequentially rotating the meander gap structure by 90°, 180°, and 270°. The simulation results show that a resonant mode with a bandwidth of about 10 MHz is supported by the cross-meandering resonator. At resonance frequency (*f* = 900 MHz), the resonator demonstrates a significant near-field enhancement effect (about 180 times that at *f* = 1 GHz). Based on a self-built shielding box test setup, the power frequency electromagnetic shielding and wireless communication signal transmission measurements are performed. The measured shielding effectiveness of the whole structure is greater than 33 dB. At the same time, the wireless communication signal can be successfully transmitted through the resonator, and the chance of being received can reach up to 100%. As a result, the proposed cross-meandering resonator combined with a cavity can effectively solve the problem of strong power frequency electromagnetic field interference and ensure the normal transmission of wireless communication signals.

## 2. Structural Design and Analysis

**Figure 1:**
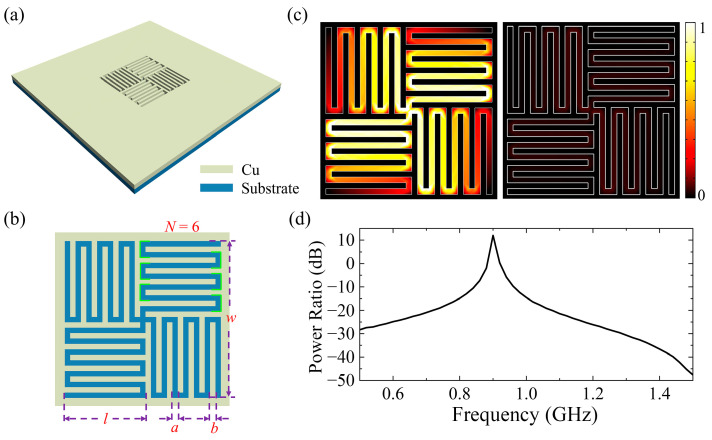
(**a**) Structural diagram of the miniaturized cross-meandering resonator. (**b**) Top view and geometric parameters of the resonator. The length and width of meandering gaps, the width of the meandering strips, and the number of corners and the size of the resonant element are denoted by *l*, *a*, *b*, *N* and *w*, respectively. Here, the number of corners, which refers to the number of bends in the resonator branches (green line marks), is *N* = 6. (**c**) Field distributions (|*E*|) of the resonator at the frequency of 900 MHz and 1000 MHz. (**d**) Power ratios of the cross-meandering resonator based on a full-wave simulation.

To decrease the structural dimensions and ensure the polarization-insensitive characteristic, a subwavelength cross-meandering resonator with a quadruple rotational symmetry was designed, as shown in Figure 1a. The resonator comprises a metallic layer and a dielectric substrate. The material of the metallic layer is copper because of copper’s high conductivity (*σ* = 5.8 × 10^7^ S/m) [21,22]. The dielectric substrate is set to F4B265, with a relative permittivity of 2.65 [23]. The thicknesses of the metallic layer and dielectric substrate are both 1 mm. Figure 1b displays a top view of the cross-meandering resonator, which is formed by rotating the meander gap structure by 90°, 180°, and 270°, successively (Figure S1 of the Supplementary Materials). The resonator offers a long induction current path within a limited size because its design is a good combination of folded and slotted structures [24,25], which can greatly reduce the resonator’s dimensions. Here, the number of corners, which refers to the number of bends in the resonator branches (green line marks in Figure 1b), is *N* = 6. The size (*w* = 29 mm) of the resonant element is only 1/11 of the free-space wavelength, which is much smaller than that of previous designs [9,11,13,19,26,27,28]. To investigate the electromagnetic properties of the cross-meandering resonator, full-wave numerical simulations were carried out based on the finite element method. The structure was excited by a *y*-polarized electric point dipole 30 mm above the resonator. A schematic diagram of the simulation calculation is displayed in Figure S2a of the Supplementary Materials. The scattering boundary conditions and perfectly matched layers are applied on the sides of the structure. When the geometric parameters of the resonator are *a* = *b* = 1 mm, *N* = 6, and *l* = 15 mm, the electric field distributions (|*E*|) at the electromagnetic frequency of *f* = 900 MHz and *f* = 1000 MHz are as shown in Figure 1c. It can be seen that the field energy is mainly localized in the meander gap of the resonator at *f* = 900 MHz. The power ratio of the cross-meandering resonator is calculated by the formula *G* = 10 × log_10_ (*P*_1/_*P*_0_), where *P*_1_ and *P*_0_ represent the radiant energy flow with and without the resonator, respectively. The calculated power ratios at different frequencies are displayed in Figure 1d. A resonance mode at around *f* = 900 MHz is observed, and the 3 dB bandwidth is about 10 MHz. Because of the significant near-field interaction (about 180 times that at *f* = 1 GHz) [29,30], the power ratio at the resonance frequency reaches 12 dB. When the vertical distance between the electric dipole and the resonator changes, the calculated power ratios at different frequencies are as shown in Figure S2b of the Supplementary Materials. It can be seen that the power ratio at the resonance frequency reduces with the increase of the vertical distance. The equivalent circuit model (ECM) is used to analyze the resonant properties of the cross-meandering resonator in theory. The transmission spectrum of the resonator array is shown in Figure S3 of the Supplementary Materials. There is a resonant peak at *f* = 900 MHz in the transmission spectrum, which also verifies that the resonator can effectively transmit a 900 MHz wireless communication signal.

**Figure 2:**
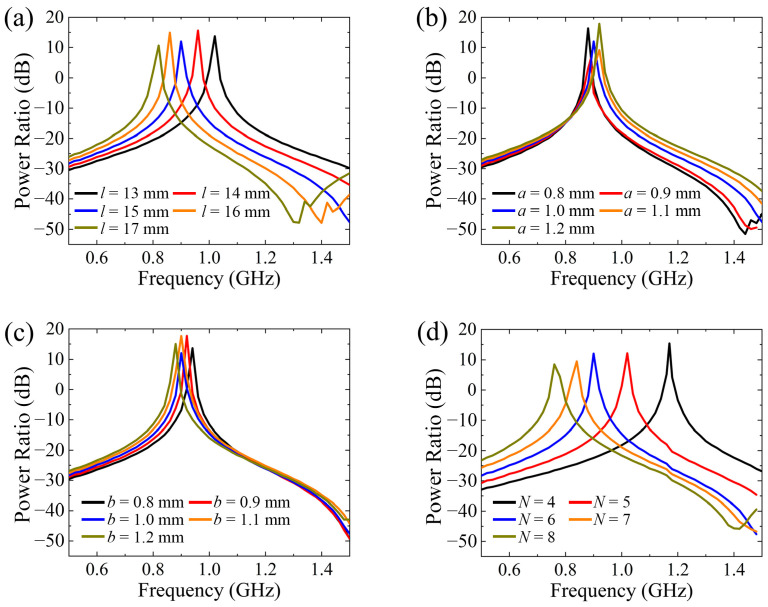
Power ratios of the resonator at different (**a**) lengths of meandering gaps, (**b**) widths of meandering gaps, (**c**) widths of meandering strips, and (**d**) number of corners.

Next, the properties of the cross-meandering resonator with changes to its geometric parameters are investigated in detail. The calculated power ratios as functions of the length (*l*) and width (*a*) of meandering gaps, the width of the meandering strips (*b*), and the number of corners (*N*) are displayed in Figure 2a–d. According to the transmission-line theory [31,32,33,34], the resonance frequency is inversely correlated with the equivalent inductance and capacitance of the cross-meandering resonator [35,36]. As shown in Figure 2a, with the increase of the length of the meandering gaps, the resonance frequency shifts from 1.02 GHz to 0.81 GHz due to the increment of the equivalent inductance of the resonator. As the width of the meandering gaps increases from *a* = 0.8 mm to *a* = 1.2 mm, the resonance frequency rises by 0.04 GHz (Figure 2b), because the equivalent capacitance of the resonator reduces slightly. Correspondingly, with the growth of the meandering strips’ width, the resonance frequency declines a little owing to the additive equivalent capacitance (Figure 2c). When the number of corners changes from *N* = 4 to *N* = 8, the resonance frequency of the structure decreases dramatically because of the incremental equivalent inductance, as depicted in Figure 2d. In comparison, the length of the meandering gaps and the number of corners have more influence on the resonance frequency of the cross-meandering resonator. Overall, the resonance frequency of the structure can be flexibly adjusted by varying the geometry parameters, so that the designed resonator can be readily adapted to different frequency ranges to meet diverse application demands.

In order to verify the power frequency electromagnetic field shielding effect of the cross-meandering resonator, the power frequency electric and magnetic field changes after passing through the resonator are simulated. Herein, five sides of a permalloy layer and one side of a copper layer form a hollow cube box (160 mm × 160 mm × 160 mm). The thicknesses of permalloy layer and copper layer are both 1 mm. The conductivity and relative permeability of the permalloy material are *σ* = 8.475 × 10^5^ S/m and *μ*_r_ = 10^5^, respectively. The center of the copper layer is the cross-meandering resonator, and the parameters are *w* = 29 mm, *l* = 15 mm, *a* = 1 mm, *b* = 1 mm. A wire with a diameter of 30 mm is placed 50 mm from the resonator. The voltage applied on the wire is 110 kV. At *f* = 50 Hz, because the element size (29 mm) is much smaller than the corresponding wavelength (6 × 10^6^ m), the cross-meandering resonator is in a non-resonant state. By utilizing the high conductivity and relative permeability of the material, the structure can realize the power frequency electromagnetic field shielding.

**Figure 3:**
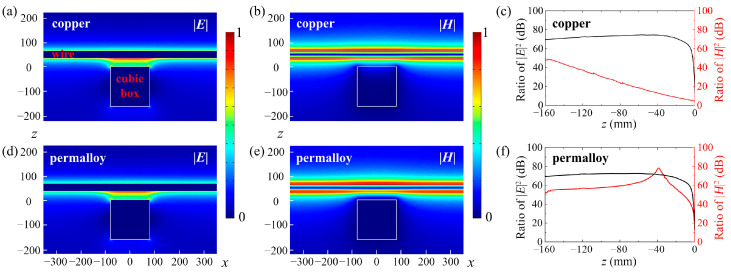
(**a**) Electric field (|*E*|) and (**b**) magnetic field (|*H*|) distributions when the material of the resonator layer is copper (white fonts). The “wire” and “cubic box” refer to the positions where the wire and cubic box are located in the simulation calculation. (**c**) Calculated ratios of |*E*|^2^ and |*H*|^2^ at the resonator layer composed of copper. (**d**) Electric field (|*E*|) and (**e**) magnetic field (|*H*|) distributions when the material of the resonator layer is permalloy (white fonts). (**f**) Calculated ratios of |*E*|^2^ and |*H*|^2^ at the resonator layer composed of permalloy.

The simulated electric field and magnetic field distributions are displayed in Figure 3a,b. It is observed that the electric field intensity inside the cubic box decreases sharply, while the magnetic field intensity is relatively gradually decreased. Taking the distributions of electric field and magnetic field without the cubic box as a reference, the calculated ratios of |*E*|^2^ and |*H*|^2^ are as shown in Figure 3c. The shielding effect of the resonator composed of copper material on the electric field (black line) is better than that on the magnetic field (red line). At *z* < −5 mm, the shielding’s effectiveness on the electric field is greater than 60 dB. To improve the ‘s effect on the magnetic field, the material of the resonator layer can be changed to permalloy. The simulated results are displayed in Figure 3d–f. It can be seen that the shielding effect of the resonator on the power frequency magnetic field has been significantly improved. The shielding effectiveness on both the electric field and magnetic fields exceeds 50 dB at *z* < −13 mm.

## 3. Experiments and Results

**Figure 4:**
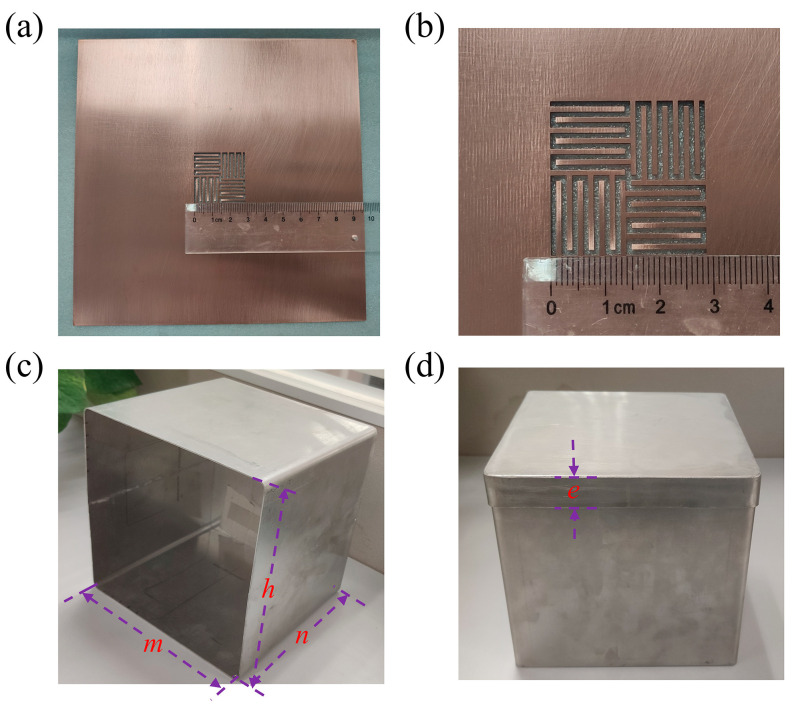
(**a**) Photograph of the cross-meandering resonator sample. (**b**) Zoomed-in view of the resonator. (**c**) Shielding box made of permalloy. The length, width and height of the shielding box are represented by *m*, *n* and *h* respectively. (**d**) Shielding box and lid made of permalloy. The width of the curled edge of the lid is represented by *e*.

To experimentally verify the functionality of the proposed cross-meandering resonator, the structure is etched in a 1 mm thick copper film using wire electrical discharge machining (WEDM). Figure 4a shows a photograph of the fabricated sample. The overall size of the sample is about 164 mm × 164 mm (~0.49*λ*_0_ × 0.49*λ*_0_). A detailed size comparison can be seen in Table S1 of the Supplementary Materials. A zoomed-in view of the resonator is displayed in Figure 4b. The measured geometrical parameters of the resonator are as follows: *w* = 28.95 mm, *l* = 14.95 mm, *a* = 0.95 mm, *b* = 1.05 mm. To test the shielding efficiency and wireless signal transmission of the sample, we customized an open side shielding box and a rolled-up lid, as depicted in Figure 4c,d. Here, the box and lid are made of permalloy, due to its high permeability at the power frequency, and the thicknesses are both 1 mm. The dimensions of the shielding box and lid are *m* = *h* = *n* = 160 mm, *e* = 10 mm.

**Figure 5:**
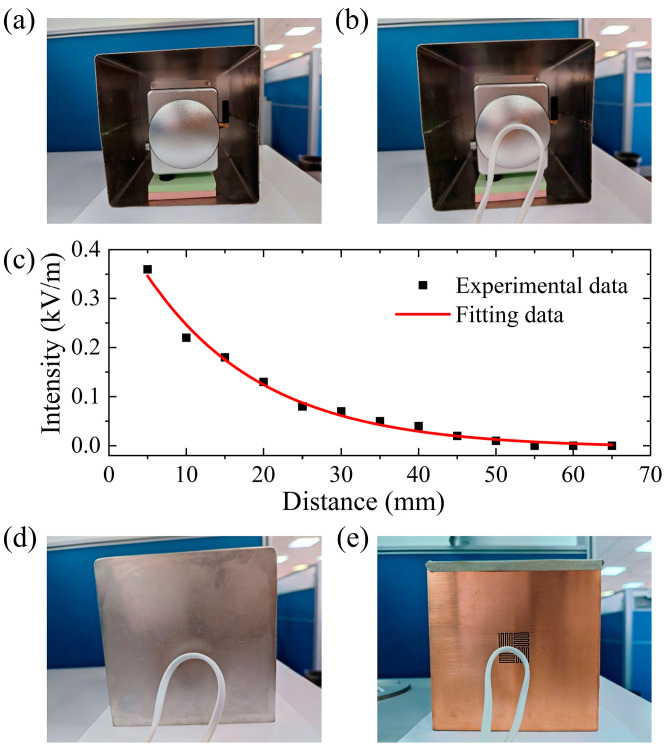
(**a**) Power frequency electric field shielding performance measurement setup. (**b**) Measured photograph when the electric wire is close to the sensor. (**c**) Measured electric field intensities at different distances (black dots). The red line indicates the fitting curve. Measured photographs when the electric wire is close to the (**d**) permalloy lid and (**e**) cross-meandering resonator sample.

To measure the power frequency electric field intensity, a MEMS AC/DC electric field sensor (JDC-W01) was brought from Beijing Zhongke Feilong Sensing Technology Company Limited (Beijing, China). In the experiment, the electric field sensor was supported in the open-sided shielding box by a stack of sticky notes, as shown in Figure 5a. An electric wire was used as the emission source of power frequency (50 Hz) electromagnetic field. When the whole device was in an open environment (Figure 5b), we measured electric field intensities, which changed with the change of the distance between the electric wire and sensor, as are displayed by the black dots in Figure 5c. The red line in Figure 5c is a fitting curve based on the equation *I* = *I*_0_ + *Ae*^−D/*t*^, where *I* and *D* represent the intensity and distance, respectively. The fitting coefficients are *I*_0_ = −0.005, *A* = 0.491, and *t* = 14.948. This indicates that the electric field intensity decreases exponentially with the distance.

Then, the permalloy lid and cross-meandering resonator sample were tightly connected with the cubic box. To achieve good electrical contact, we first pasted the box and resonator surface together with an electromagnetic shielding sealing strip, and then covered the joint with a conductive cloth tape. In the follow-up experiments, we put the electric wire close to the permalloy lid and cross-meandering resonator sample, as shown in Figure 5d,e. Table 1 shows the results of multiple measurements in three conditions. It is obviously observed that the cross-meandering resonator sample and the permalloy lid both effectively block the transmission of a power frequency electromagnetic field, because the electric field intensity in the shielding box is less than the minimum that the sensor can measure (10 V/m). According to the equation *SE* = 20 × log_10_(*E*_0_/*E*_s_) [22], in which *E_0_* and *E_s_* represent the electric field intensities in open environment and covered with the resonator sample, respectively, the measured shielding effectiveness can exceed 33 dB, which is comparable to that observed in previous works [26,28] (Table S1 of the Supplementary Materials). Hence, the proposed cross-meandering resonator could find potential applications in power frequency electromagnetic field shielding of UHV power grids.

**Figure 6:**
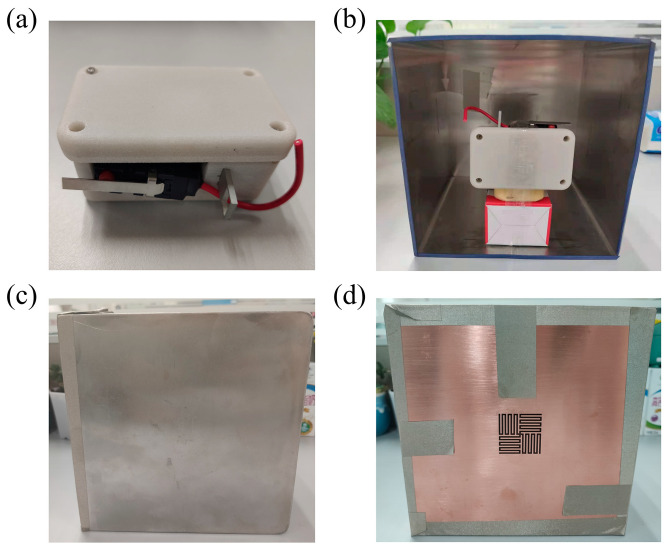
(**a**) Photograph of the air pressure sensor. Measured photographs when the whole device (**b**) in an open condition, (**c**) with the permalloy lid on, and (**d**) covered with the cross-meandering resonator sample.

In addition to testing the shielding performance of the power frequency electromagnetic field, we also studied the transmission of wireless communication signals by the cross-meandering resonator sample. Herein, an air pressure sensor (Figure 6a) composed of a MEMS piezoresistive pressure sensor, temperature sensor, and integrated narrow-band Internet of Things (NB-IoT) communication module was independently developed. The sensor can send barometric data to the software platform via a 900 MHz wireless communication signal. By utilizing the low power technology and temperature compensation algorithm, the air pressure sensor monitoring system has the advantages of low power consumption, low cost, high accuracy, and online monitoring. As in the previous experiment, the air pressure sensor is suspended in the open-sided shielding box, as depicted in Figure 6b. Three controlled experiments—the whole device in an open condition (Figure 6b), with the permalloy lid on (Figure 6c) and covered with the cross-meandering resonator sample (Figure 6d) were also performed. It should be noted that the sample in Figure 6d is the same as that in Figure 5e, and the difference in color is caused by different ambient light. If a barometric value is displayed in the online post-processing platform, the wireless communication signal transmitted by the air pressure sensor passed smoothly through the cross-meandering resonator sample and was successfully received by the NB-IoT base station. In this case, we used the symbol of “√” to record the result. Conversely, if no data was updated, the signal was not received and the symbol of “×” was recorded. The measured results at three conditions are shown in Table 2.

**Figure 7:**
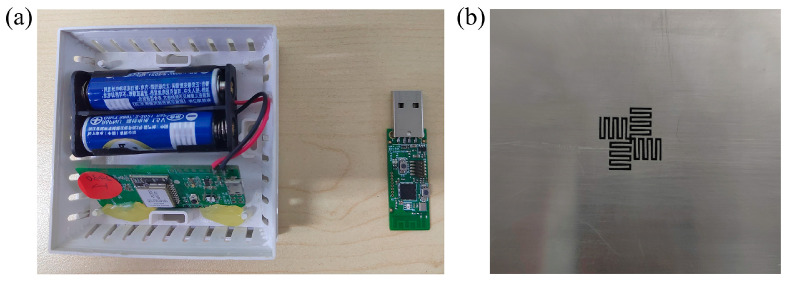
(**a**) Photograph of the 2.4 GHz wireless communication module (left part) and the signal receiving module (right part). (**b**) Photograph of the resonator sample with a resonance frequency of 2.4 GHz.

When the shielding box was open, the wireless communication signal was effectively received by the NB-IoT base station. The probability is not 100% because the communication signal transmitted between the self-developed sensor and the base station was unstable. As a contrast, the wireless communication signal was completely blocked by the permalloy lid. When the shielding box was covered by the cross-meandering resonator sample, the chance of the signal being received by the NB-IoT base station was about 53%. The low probability was attributed to the instability of the communication signal and resonant frequency deviations. To remove the instability of the communication signal between the sensor and NB-IoT base station, a 2.4 GHz wireless communication module (left part in Figure 7a) and the signal receiving module (right part in Figure 7a) were brought from Shenzhen Ruidilai Technology Company Limited (Shenzhen, China). By adjusting the structural parameters of the cross-meandering resonator, the resonance frequency shifted to near 2.4 GHz. The fabricated resonator sample composed of the permalloy material is displayed in Figure 7b. Then the communication module was placed in the shielding box, and the receiving module was inserted into a computer. The distance between the communication module and receiving module was about 2 m. Consistent with the testing method of Figure 6, the controlled experiments were carried out, and the results are shown in Table 3. It can be seen that the 2.4 GHz wireless communication signal passed perfectly through the resonator and was received by the receiving module, with a probability of 100%. Therefore, the above experimental results fully verify that the proposed cross-meandering resonator can not only shield the power frequency electromagnetic field but also allow wireless communication signal transmission, which has not been explored before (Table S1 of the Supplementary Materials).

## 4. Conclusions

In summary, by utilizing a subwavelength cross-meandering resonator (0.49*λ*_0_ × 0.49*λ*_0_), power frequency electromagnetic field shielding and wireless communication signal transmission were successfully demonstrated. The resonator had a quadruple rotational symmetry, and its element size (~*λ*_0_/11) is much smaller than that of previous works [9,11,13,19,26,27,28]. Due to the significant near-field enhancements of |*E*|^2^ at *f* = 900 MHz, which is about 180 times that at *f* = 1 GHz, a resonant mode with a power ratio of 12 dB was supported by the cross-meandering resonator. Based on the self-made shielding box experimental setup, the power frequency electromagnetic field shielding and wireless sensing communication performance of the resonator sample were tested. The measured shielding effectiveness could exceed 33 dB, and the probability of a wireless communication signal being received could reach up to 100%. Therefore, the proposed cross-meandering resonator simultaneously realized the goals of power frequency electromagnetic shielding and transmission of wireless sensing communication signals. By integrating the dual-functional resonator and MEMS devices, we expect to solve many of the physical problems faced by wireless sensors in strong electromagnetic environments and greatly promote the development of MEMS wireless sensing technology adapted to strong electromagnetic field environments.

## Figures and Tables

**Table 1:** Measured electric field intensities at three conditions (kV/m).

	1	2	3	4	5
Open	0.446	0.442	0.452	0.450	0.448
Permalloy lid	<0.01	<0.01	<0.01	<0.01	<0.01
Resonator sample	<0.01	<0.01	<0.01	<0.01	<0.01

**Table 2:** Experimental data of 900 MHz wireless communication at multiple measurements. The numbers in the table represent the times of measurement.

	1	2	3	4	5	6	7	8	9	10	11	12	13	14	15	Probability
Open	√	√	√	√	√	√	√	√	√	√	√	√	√	√	√	93%
	√	√	×	√	√	√	×	√	√	√	√	√	√	√	√	
Permalloy lid	×	×	×	×	×	×	×	×	×	×	×	×	×	×	×	0%
Resonator sample	√	×	×	×	√	√	×	√	×	√	×	√	√	√	×	53%

**Table 3:** Experimental data of 2.4 GHz wireless communication at multiple measurements.

	1	2	3	4	5	6	7	8	9	10	11	12	13	14	15	Probability
Open	√	√	√	√	√	√	√	√	√	√	√	√	√	√	√	100%
	√	√	√	√	√	√	√	√	√	√	√	√	√	√	√	
Resonator sample	√	√	√	√	√	√	√	√	√	√	√	√	√	√	√	100%

## Data Availability

The data presented in this study are available on request from the corresponding author.

## Supplementary Materials

The following supporting information can be downloaded at: https://www.mdpi.com/article/10.3390/s24175615/s1, Figure S1: Composition method of the cross-meandering resonator; Figure S2: Schematic diagram of the simulation calculation and power ratios at different vertical distances; Figure S3: Calculated transmission spectrum of the resonator array by using the equivalent circuit model; Table S1: Comparison of the proposed resonator with other works.

## References

[1] Shi C., Andino-Pavlovsky V., Lee S.A., Costa T., Elloian J., Konofagou E.E., Shepard K.L. (2021). Application of a sub–0.1-mm^3^ implantable mote for in vivo real-time wireless temperature sensing. Sci. Adv..

[2] Modalavalasa S., Sahoo U.K., Sahoo A.K., Baraha S. (2021). A review of robust distributed estimation strategies over wireless sensor networks. Signal Process..

[3] Kruželák J., Kvasničáková A., Hložeková K., Hudec I. (2021). Progress in polymers and polymer composites used as efficient materials for EMI shielding. Nanoscale Adv..

[4] Zakaria N.A., Sudirman R., Jamaluddin M.N. Electromagnetic interference effect from power line noise in electrocardiograph signal using faraday cage. Proceedings of the 2008 IEEE 2nd International Power and Energy Conference.

[5] Liu P., Wang P., Deng Y., Wu F., Dai X., Wang Y. Preparation of silver-coated copper electromagnetic shielding coating and its application in power frequency electromagnetic field. Proceedings of the IOP Conference Series: Earth and Environmental Science.

[6] Li T.-W., Li D., Li E.-P. A novel FSS structure with high selectivity and excellent angular stability for 5G communication radome. Proceedings of the 2017 10th Global Symposium on Millimeter-Waves.

[7] Chakraborty U., Chatterjee S., Chowdhury S.K., Sarkar P.P. (2011). A comact microstrip patch antenna for wireless communication. Prog. Electromagn. Res. C.

[8] Wang H., Qu S., Wang J., Yan M., Zheng L. (2020). Dual-band miniaturised FSS with stable resonance frequencies of 3.4/4.9 GHz for 5G communication systems applications. IET Microw. Antennas Propag..

[9] Smith T., Gothelf U., Kim O.S., Breinbjerg O. (2013). An FSS-backed 20/30 GHz circularly polarized reflectarray for a shared aperture L-and Ka-band satellite communication antenna. IEEE Trans. Antennas Propag..

[10] Ma Y., Wu W., Yuan Y., Zhang X., Yuan N. (2018). A Wideband FSS Based on Vias for Communication Systems. IEEE Antennas Wirel. Propag. Lett..

[11] Mantash M., Kesavan A., Denidni T.A. (2017). Beam-tilting endfire antenna using a single-layer FSS for 5G communication networks. IEEE Antennas Wirel. Propag. Lett..

[12] Hong T., Wang M., Peng K., Zhao Q., Gong S. (2020). Compact Ultra-Wide Band Frequency Selective Surface with High Selectivity. IEEE Trans. Antennas Propag..

[13] Bindu K., Chopra R., Kumar G. Low Cost broadband stacked circular microstrip antenna. Proceedings of the 2017 IEEE International Conference on Antenna Innovations & Modern Technologies for Ground, Aircraft and Satellite Applications (iAIM).

[14] Boukarkar A., Lin X.Q., Jiang Y., Yang X.F. (2018). A Compact Frequency-Reconfigurable 36-States Patch Antenna for Wireless Applications. IEEE Antennas Wirel. Propag. Lett..

[15] Zidan M.S. (2019). Design and Analysis of Frequency-Reconfigurable Microstrip Antenna using Multiple Parasitic Patches. IOP Conf. Ser. Mater. Sci. Eng..

[16] Serup D.E., Williams R.J., Zhang S., Pedersen G.F. Shared aperture dual S-and X-band antenna for nano-satellite applications. Proceedings of the 2020 14th European conference on antennas and propagation (EuCAP).

[17] Johnson A.D., Manohar V., Venkatakrishnan S.B., Volakis J.L. (2020). Low-Cost S-Band Reconfigurable Monopole/Patch Antenna for CubeSats. IEEE Open J. Antennas Propag..

[18] Sun M., Zhang Z., Zhang F., Chen A. (2019). L/S Multiband Frequency-Reconfigurable Antenna for Satellite Applications. IEEE Antennas Wirel. Propag. Lett..

[19] Akhila P., Kumar S.A., Shanmuganantham T. Antenna design for nanosatellite payload communication system. Proceedings of the 2020 IEEE International Conference on Electronics, Computing and Communication Technologies (CONECCT).

[20] Ramahatla K., Mosalaosi M., Yahya A., Basutli B. (2022). Multiband Reconfigurable Antennas for 5G Wireless and CubeSat Applications: A Review. IEEE Access.

[21] Koroglu S., Umurkan N., Kilic O. Experimental performance investigation of double-layer shields at power frequency magnetic shielding. Proceedings of the 2008 Power Quality and Supply Reliability Conference.

[22] Geetha S., Satheesh Kumar K.K., Rao C.R.K., Vijayan M., Trivedi D.C. (2009). EMI shielding: Methods and materials-A review. J. Appl. Polym. Sci..

[23] Xiao D., Liu Y., Li S., Sun O. An Improved Design of Center-Fed SIW Slot Dual-Layered Substrate Antenna. Proceedings of the 2022 International Conference on Microwave and Millimeter Wave Technology (ICMMT).

[24] Foudazi A., Hassani H.R., Nezhad S.M.A. (2012). Small UWB Planar Monopole Antenna with Added GPS/GSM/WLAN Bands. IEEE Trans. Antennas Propag..

[25] Wu M.-T., Chuang M.-L. (2015). Multibroadband Slotted Bow-Tie Monopole Antenna. IEEE Antennas Wirel. Propag. Lett..

[26] Sivasamy R., Moorthy B., Kanagasabai M., Samsingh V.R., Alsath M.G.N. (2017). A wideband frequency tunable FSS for electromagnetic shielding applications. IEEE Trans. Electromagn. Compat..

[27] Gurrala P., Oren S., Liu P., Song J., Dong L. (2017). Fully conformal square-patch frequency-selective surface toward wearable electromagnetic shielding. IEEE Antennas Wirel. Propag. Lett..

[28] Syed I.S., Ranga Y., Matekovits L., Esselle K.P., Hay S.G. (2014). A single-layer frequency-selective surface for ultrawideband electromagnetic shielding. IEEE Trans. Electromagn. Compat..

[29] Li M., Zhong B.G., Cheung S.W. (2019). Isolation Enhancement for MIMO Patch Antennas Using Near-Field Resonators as Coupling-Mode Transducers. IEEE Trans. Antennas Propag..

[30] Li K., Hogan N.J., Kale M.J., Halas N.J., Nordlander P., Christopher P. (2017). Balancing Near-Field Enhancement, Absorption, and Scattering for Effective Antenna–Reactor Plasmonic Photocatalysis. Nano Lett..

[31] Zhang M., Zhang F., Ou Y., Cai J., Yu H. (2019). Broadband terahertz absorber based on dispersion-engineered catenary coupling in dual metasurface. Nanophotonics.

[32] Varkani A.R., Firouzeh Z.H., Nezhad A.Z. (2018). Equivalent circuit model for array of circular loop FSS structures at oblique angles of incidence. Iet Microw. Antennas Propag..

[33] Mahdi R., Hamid R., Ali A. (2018). Multilayer graphene-based metasurfaces: Robust design method for extremely broadband, wide-angle, and polarization-insensitive terahertz absorbers. Appl. Opt..

[34] Barzegar-Parizi S. (2018). Realization of wide-angle and wideband absorber using metallic and graphene-based metasurface for mid-infrared and low THz frequency. Opt. Quantum Electron..

[35] Yan M., Qu S., Wang J., Ma H., Zhang J., Wang W., Zheng L., Yuan H. (2015). A single layer ultra-miniaturized FSS operating in VHF. Photonics Nanostructures Fundam. Appl..

[36] Sheokand H., Ghosh S., Singh G., Saikia M., Srivastava K.V., Ramkumar J., Anantha Ramakrishna S. (2017). Transparent broadband metamaterial absorber based on resistive films. J. Appl. Phys..

