# Supplementary material for: Dual-Functional Cross-Meandering Resonator for Power Frequency Electromagnetic Shielding and Wireless Sensing Communication
Source: Sensors (Basel). 2024 Aug 29;24(17):5615. doi: 10.3390/s24175615 (PMC11398102; doi:10.3390/s24175615)
Supplement: Supplementary file 1 [file sensors-24-05615-s001.zip › sensors-3088006-supplementary.pdf]

Supplementary Material

Dual-Functional Cross-Meandering Resonator for Power Frequency Electromagnetic Shielding and Wireless Sensing Communication

Fengyuan Gan [1,2,*], Xiangshuo Shang [2], Xuelei Yang [2], Shuo Li [3], Yi Zhou [2] and Wei Li [2,*]

[1] School of Physical Science and Technology, Southwest University, Chongqing 400715, China
[2] National Key Laboratory of Materials for Integrated Circuits, Shanghai Institute of Microsystem and Information Technology, Chinese Academy of Sciences, Shanghai 200050, China; sxs3830@163.com (X.S.); yxl1231@mail.ustc.edu.cn (X.Y.); yizhou_scarlett@126.com (Y.Z.)
[3] College of Energy and Mechanical Engineering, Shanghai University of Electric Power, Shanghai 201306, China; shuoli0107@gmail.com
* Correspondence: ganfengyuan@swu.edu.cn (F.G.); waylee@mail.sim.ac.cn (W.L.)

The composition method of the cross-meandering resonator is as follows. Firstly, the meander gap structure (Figure S1a) is assembled to achieve a greater effective length within a limited size. Then, the cross-meandering resonator (Figure S1b) is formed by rotating the meander gap structure by 90°, 180°, and 270° successively. The resonator obtains the long induction current path by a good combination of the folded and slotted structure [24,25], which can greatly reduce the resonator dimension.

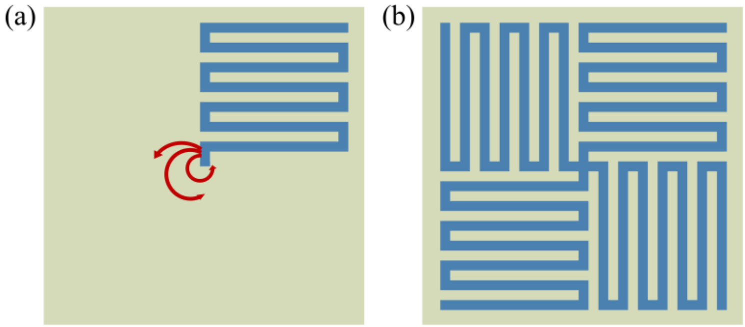

Figure S1. (a) Top view of the meander gap structure. (b) Top view of the cross-meandering resonator.

Full-wave numerical simulations are carried out in COMSOL Multiphysics 6.0 (trial version). In the simulation, the electric point dipole, which is an excitation light source of the software, is 30 mm above the cross-meandering resonator. The schematic diagram of the simulation calculation is displayed in Figure S2a. When the vertical distance between the electric dipole and the resonator changes, the calculated power

ratios at different frequencies are shown in Figure S2b. It can be seen that the position of the electric dipole does affect the performance of the cross-meandering resonator. With the increase of the vertical distance, the radiant energy flow at resonance frequency reduces.

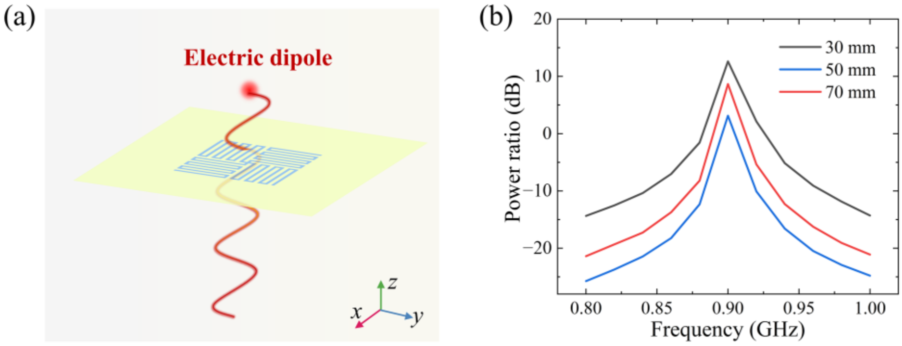

Figure S2. (a) Schematic diagram of the simulation calculation of the cross-meandering resonator. (b) Power ratios at different vertical distances.

The equivalent circuit model (ECM) is used to analyze the resonant properties of the cross-meandering resonator in theory. The transmission spectrum of the resonator array is shown in Figure S3. Here, the values of the equivalent inductance, equivalent capacitance, and characteristic impedance of the circuit model are determined as L = 90.082 nH, C = 300.7 pF, Z_0 = 377 Ω, and $Z_d = Z_0/\sqrt{\varepsilon_r}$=231 Ω. There is a resonant peak at f = 900 MHz in the transmission spectrum, which also verifies that the resonator can effectively transmit the 900 MHz wireless communication signal.

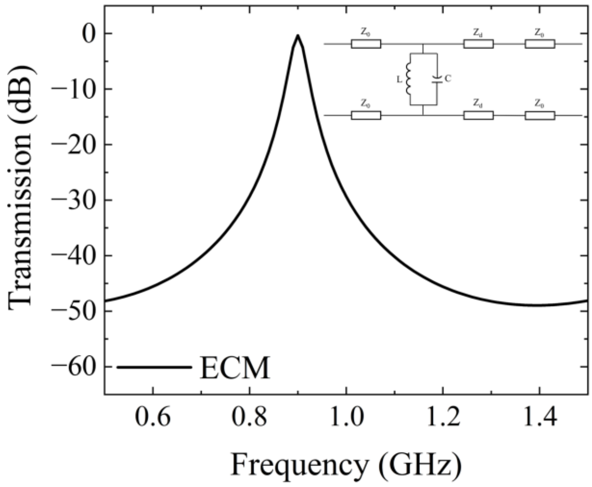

Figure S3. Calculated transmission spectrum of the resonator array by using the equivalent circuit model.

The comparison of the proposed resonator sample with other works has been shown in Table S1. It is observed that the element size of the cross-meandering resonator is much smaller than that in Refs [9], [11], [13], [19] and [26-28]. Although the element size in our work is slightly greater than that in Ref [10], the total size is much smaller than that of the array structure in this work. It should be noted that the total size of the resonator sample in our work can be further compressed to about $0.3\lambda_0 \times 0.3\lambda_0$ in theory. For the electromagnetic field shielding, some works use composite or nanocoating materials to improve the shielding effectiveness. When utilizing the composite or nanocoating shielding materials, however, the structures are difficult to fabricate experimentally and the wireless communication frequency band cannot be flexibly controlled. Besides, the frequency selective surfaces are also designed to shield electromagnetic fields [26-28]. The shielding effectiveness of the proposed resonator is comparable to that of Refs [26] and [28]. More importantly, these works [9-11,13,19,26-28] did not simultaneously demonstrate the electromagnetic shielding and the wireless communication signal transmission in the experiment.

Table S1. Comparison of the proposed resonator with other works

Works	Resonant element size	Total size of structure	Shielding Effectiveness	Wireless communication
Proposed	$\lambda_0/11$	$0.49\lambda_0 \times 0.49\lambda_0$ (164×164 mm^2)	> 33 dB	√
Ref [10]	$\lambda_0/16$	$2.57\lambda_0 \times 2.57\lambda_0$	/	√
Ref [9]	$\lambda_0/3$	$26.7\lambda_0 \times 26.7\lambda_0$	/	√
Ref [11]	$\lambda_0/4$	$13\lambda_0 \times 9\lambda_0$	/	√
Ref [13]	$\lambda_0/1.07$	$0.94\lambda_0 \times 0.94\lambda_0$	/	√
Ref [19]	$\lambda_0/5.67$	$0.33\lambda_0 \times 0.33\lambda_0$	/	√
Ref [26]	$\lambda_0/1.96$	$6.6\lambda_0 \times 6\lambda_0$	~ 33.2 dB	/
Ref [27]	$\lambda_0/5$	- (array)	> 15 dB	/
Ref [28]	$\lambda_0/3.12$	$11.5\lambda_0 \times 7.4\lambda_0$	> 40 dB	/
